# Novel investigations in retinoic-acid-induced cleft palate about the gut microbiome of pregnant mice

**DOI:** 10.3389/fcimb.2022.1042779

**Published:** 2022-12-15

**Authors:** Yijia Wang, Jing Chen, Xiaotong Wang, Cui Guo, Xia Peng, Ying Liu, Tianli Li, Juan Du

**Affiliations:** ^1^ Laboratory of Orofacial Development, Capital Medical University School of Stomatology, Beijing, China; ^2^ Laboratory of Molecular Signaling and Stem Cells Therapy, Capital Medical University School of Stomatology, Beijing, China; ^3^ Molecular Laboratory for Gene Therapy and Tooth Regeneration, Capital Medical University School of Stomatology, Beijing, China; ^4^ Beijing Key Laboratory of Tooth Regeneration and Function Reconstruction, Capital Medical University School of Stomatology, Beijing, China; ^5^ Department of Geriatric Dentistry, Capital Medical University School of Stomatology, Beijing, China; ^6^ Laboratory of Oral Microbiology, Capital Medical University School of Stomatology, Beijing, China

**Keywords:** cleft palate, retinoic acid, gut microbiome, lactobacillus, metabolism

## Abstract

**Introduction:**

Cleft palate (CP) is one of the most common congenital birth defects in the craniofacial region, retinoic acid (RA) gavage is the most common method for inducing cleft palate model. Although several mechanisms have been proposed to illuminate RA-induced cleft palate during embryonic development, these findings are far from enough. Many efforts remain to be devoted to studying the etiology and pathogenesis of cleft palate. Recent research is gradually shifting the focus to the effect of retinoic acid on gut microbiota. However, few reports focus on the relationship between the occurrence of CP in embryos and gut microbiota.

**Methods:**

In our research, we used RA to induce cleft palate model for E10.5 the feces of 5 RA-treated pregnant mice and 5 control pregnant mice were respectively metagenomics analysis.

**Results:**

Compared with the control group, Lactobacillus in the gut microbiome the RA group was significantly increased. GO, KEGG and CAZy analysis of differentially unigenes demonstrated the most abundant metabolic pathway in different groups, lipopolysaccharide biosynthesis, and histidine metabolism.

**Discussion:**

Our findings indicated that changes in the maternal gut microbiome palatal development, which might be related to changes in Lactobacillus and These results provide a new direction in the pathogenesis of CP induced by RA.

## Introduction

Cleft palate (CP) is one of the most common congenital birth defects in the craniofacial region, with an average occurrence of 1/1000 newborns around the world (Wang et al., 2019a). It is universally acknowledged that CP has a connection with genetic background and environmental factors. As similarities in palatogenesis between humans and mice have been noted, the mouse model considerably contributes to the study of the etiology of cleft palate in humans (Peng et al., 2020).

Nowadays, many studies have shown that retinoic acid (RA) gavage is the most common method for inducing cleft palate model except in knockout mice (Abbott and Birnbaum, 1990; Degitz et al., 1998). RA is one of the crucial trace elements in embryonic development, which plays an essential role in the regulation of morphology, cell proliferation and differentiation, and the production of extracellular matrix (Wang and Kirsch, 2002). The proliferation of palatal mesenchymal cells was inhibited by RA at embryo days (E) 10.5, resulting in cleft palate and no apoptosis of palatal epithelial cells (Goulding and Pratt, 1986). Although several mechanisms have been proposed to illuminate RA-induced cleft palate during embryonic development, these findings are focused on the changes in the embryo palate. As RA was first to work on pregnant mice which subsequently influence the embryos, further efforts about RA and the pregnant mice on the etiology and pathogenesis of cleft palate need to be studied. Current research is gradually shifting the focus to the effect of retinoic acid on gut microbiota. The “microbiota” consists of microbiota communities on the mucosal surface and lumen of the respiratory tract, gastrointestinal (GI) tract, urinary tract, and reproductive tract. The GI tract has the greatest density of microbiota, defined as the “gut microbiota” (Alipour et al., 2016; Sililas et al., 2021). Gut microbiota is essential for digesting food, producing short-chain fatty acids, synthesizing vitamins, and protecting the mucosal. To a large extent, host metabolism and immune response were due to the interaction between host cells and the gut microbiota (Quan et al., 2020). The intestinal microbiota imbalance was mainly manifested by an increase in harmful bacteria and a decrease in beneficial bacteria. Microbiota imbalances were associated with several underlying diseases, for instance, metabolic syndrome, allergic diseases, some kinds of cancer, and neurological diseases (Schroeder and Bäckhed, 2016; Wang et al., 2019b; Han et al., 2020). As an important modulator of innate immune cells, RA played an essential role in defending the intestinal immune system (Czarnewski et al., 2017). Research on Alzheimer’s disease has found that RA could influence gut by regulating commensal microbiota. These in turn could also interfere with retinoid metabolism and *via* the gut-brain axis furthermore with Alzheimer’s disease pathology within the brain (Endres, 2019).

However, the dangers of disrupted gut flora are not limited to these. Recent studies found that pregnant women with an imbalance in gut flora might be at risk not only for their health but also for their fetuses. During pregnancy, maternal gut environment could finetune energy homeostasis, which was a key factor to prevent metabolic syndrome for offspring (Kimura et al., 2020). In addition, the mother’s gut flora was also a source of immunity for offspring. The neonatal mice lacking the ability to produce IgG could be protected against enterotoxigenic *Escherichia coli* infection by the mother’s natural IgG antibody to *Escherichia coli*, which was transmitted through the placenta or breast milk (Zheng et al., 2020). For the past few years, the emergence of the metagenomics could associate the microflora with genes, thereby better illustrating the mechanism of regulation of diseases by microflora disorders (Wang and Jia, 2016). For example, the maternal gut microbiome is proven to be a vital signal in developing brain neurons through microbial-regulated metabolites, which promote fetal-thalamic cortex axons (Vuong et al., 2020).

Recent studies suggest that RA disrupts the intestinal flora, while alterations in maternal intestinal flora are closely related to embryonic development (Zhou et al., 2021), but it is not known whether such a relationship exists during palatal development. Therefore, we selected E10.5 pregnant mice to construct the fetal cleft palate model by intragastric administration of RA and collected feces samples of all pregnant mice at E16.5 for metagenomics analysis. Our work aims to provide a new direction in the pathogenesis of CP induced by RA.

## Materials and methods

### Animals and sample collection

C57BL/6J mice were purchased from the Sibeifu Company (Beijing, China). Female C57BL/6J mice (age, 9-10 weeks; weight, 20-25 g) and mature male mice (age, 9-10 weeks; weight, 20-25g) mated overnight. The noon following mating with detection of a vaginal plug was designated as E0.5. Ten pregnant mice were equally divided into the control group and the RA group. The sample size calculation followed ARRIVE guidelines. As 100 mg/kg RA on pregnant mice by oral gavage was reported to have almost 100% mouse embryo CP without maternal deaths which was considered as a common dose in inducing embryo CP (Campbell et al., 2004; Dong et al., 2017; Gao et al., 2017; Peng et al., 2020), female mice at E10.5 were administered RA (100 mg/kg; Sigma-Aldrich; Merck KGaA, Darmstadt, Germany) dissolved in corn oil by oral gavage. Control mice were given an equal amount of corn oil. RA-treated and untreated pregnant mice were sacrificed *via* cervical dislocation at E16.5. All mouse experiments were taken place in Beijing Key Laboratory of Tooth Regeneration and Function Reconstruction, Capital Medical University School of Stomatology, and were approved by the Animal Care and Use Committee of the School of Stomatology, Capital Medical University (Beijing, China, permit number: KQYY-202109-006), and all experiments met the relevant regulatory standards.

To ensure that the initial flora of each group of mice was consistent, specific pathogen-free (SPF) mice were selected and raised in a barrier system. And we fed mice using sterilized litter, food, and water in the hospital’s experimental animal rooms which was SPF standard. In addition, we raised the mice individually in cages and collected the mice’s feces separately. All eligible feces samples were sent to the laboratory immediately after self-sampling of feces samples (Santiago et al., 2014), each sample was divided into 3 parts, loaded into 3 cryopreservation tubes, and stored at −80°C after overnight freezing of liquid nitrogen.

### Stereomicroscope observing and hematoxylin-eosin staining

Embryos from RA-treated mice and control mice were isolated at E16.5. Palate tissues of half of the embryos were detached by ophthalmic shears and then observed under a stereomicroscope (Olympus, Japan). Other embryos were fixed in 4% paraformaldehyde at room temperature for 24 h. All fixed samples were dehydrated by the ethanol gradient, and after 4 h of preservation of n-butanol, they were embedded with paraffin wax and sliced at 5 μm intervals to make tissue sections. After dewaxing, the structure was observed by H&E staining.

### Total RNA exteacted and DNA library construction

DNA from 5 RA samples and 5 control samples was extracted by the E.Z.N.A.^®^ Stool DNA Kit (D4015-02, Omega, Inc., USA). Sample blanks composed of unused swabs were processed through DNA extraction and tested to confirm no DNA amplicons contamination. The total DNA was eluted in 50 µl elution buffer by manufacturer’s (QIAGEN) production and measured in the PCR by LC-BIO TECHNOLOGIES (HANGZHOU) CO., LTD., Hang Zhou, Zhejiang Province. Then, DNA library was constructed by TruSeq Nano DNA LT Library Preparation Kit (FC-121-4001). First, DNA was randomly broken into 200-500 bp fragments and DNA ends were repaired. Second, an A base was added to the 3’ end of DNA fragment and a connector was added to the end of DNA fragment. At last, the ligation product was purified and amplified by PCR.

### Metagenomic sequencing and data analysis

After passing the quality inspection of the library, NovaSeq 6000 was used for high-throughput sequencing. The sequencing mode was PE150, and the sequencing kit was TruSeqNano DNA LT Library Preparation Kit-Set A(FC-121-4001). The valid data was obtained by preprocessing sequenced raw data (de-coupled and de-processed). Once quality-filtered reads were obtained, they were *de novo* assembled to construct the metagenome for each sample. All coding regions (CDS) of metagenomic contigs were predicted in order to obtain unigenes. The specific data analysis procedures were listed in **Supplementary Table 1**. Subsequently, compare unigenes with NR_mate libraries to obtain species annotation information and compare unigenes with the protein sequence of GO/KEGG/CAZy database, to obtain function annotation information. The database information was listed in **Supplementary Table 2**.

### Lactate amount test

The lactate amount in plasma, amniotic fluid, and palatal tissue was tested by CheKine™ Lactate Colorimetric Assay Kit (Abbkine Scientific Co, China). Plasma and amniotic fluid were extracted from E16.5 pregnant mice. Palatal tissue was extracted from E16.5 fetal mice. According to the weight of the palatal tissue (1 mL lactate assay buffer/0.1 g tissue), the extraction liquid was prepared and homogenized on ice, centrifuged at 12,000 g for 5 min at 4℃, then the supernatant was used for assay. The optical absorbance values were measured by a SpectraMax Paradigm microplate reader (Molecular Devices, CA, US) at 450 nm.

### Statistical analyses

For metagenomic sequencing, the statistical method of alpha diversity (observed species, Shannon, Simpson, and Chao1) was Mann Whitney U test (R, v3.4.4, p<0.05 were considered statistically significant). Beta diversity (PCA and PCoA) was analyzed by ANOSIM analysis (-1≤R ≤ 1, R value close to 1 indicated that the difference in between groups was greater than within group, and R value close to -1 indicated that there was no significant difference between and within groups; p<0.05 were considered statistically significant). The Linear discriminant analysis effect size (LEfSe) method was analyzed by pathyon. Based on the taxonomic and functional annotation of unigenes, the differential analysis was carried out at each taxonomic (family, genus, and species) or enrichment analysis (GO and KEGG) by Mann Whitney U test (R, v3.4.4, p<0.05 were considered statistically significant). As for gene expression differences, significantly different gene default thresholds was |log2(fold_change)|≥1 by Mann Whitney U test (R, v3.4.4), p<0.05 were considered statistically significant.

Statistical analyses of lactate amount test were performed with GraphPad Prism software (version 9, by MacKiev Software, Boston, MA, USA). The variance between the two groups was compared by independent two-tailed student’s t-test, p < 0.05 were considered statistically significant. The D’Agostino-Pearson test was used to verify whether the samples data came from a normal distribution. The biological replicates of metagenomic sequencing were 5 in each group. The samples of amniotic fluid, plasma, and palate tissue were separately collected from 3 different pregnant mice. And for each sample, we replicated 3 times, then calculated all of data together which get 9 points for each sample.

## Results

### The successful construction of RA-induced cleft palate model

**Figure 1:**
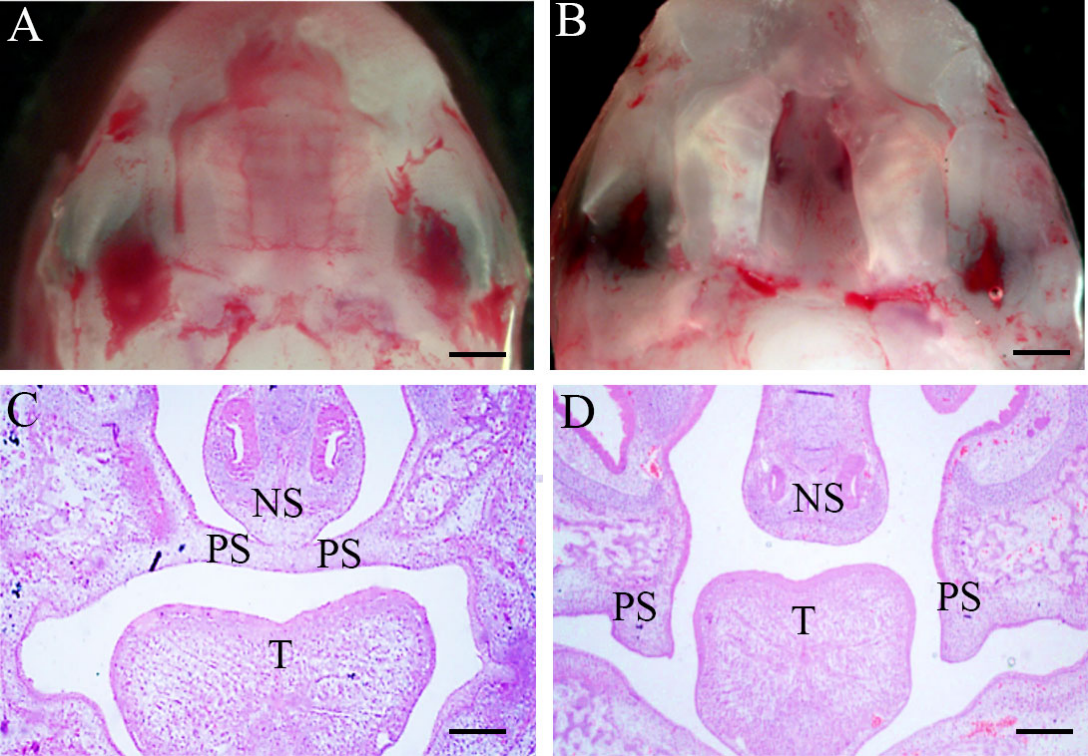
Morphology and H&E of palate shelf tissues at E16.5. **(A, C)** The palatal shelf contacted the midline and fused through the formation of the midline epithelial seam (MES) in the mid-anterior region of a control embryo. **(B, D)** Unfused, separated palatal shelf from an embryo of RA-treated mouse. **(A, B)** Morphological specimens (magnification ×100, scale bar 100 μm); **(C, D)**, H&E staining results (magnification ×40, scale bar 200 μm). PS, palatal shelf; T, tongue; NS, nasal septum; H&E, hematoxylin, and eosin.

E10.5 pregnant mice were used to construct the fetal cleft palate model by intragastric administration of RA (100 mg/kg) (Dong et al., 2017; Gao et al., 2017; Peng et al., 2020). At E16.5, when the normal palate was primarily formed, the palate of fetal mice in the control group was normally fused, and the incidence of cleft palate in the RA group was approximately 97% (38/39). In the palate shelf tissue and histological sections of control E16.5 embryos, the opposed palatal shelves had come into contact and fused during normal development (**Figures 1A, C**). In the meanwhile, RA-treated palatal shelves remained small in volume, failed to rise and fuse with the contralateral side, and were vertically oriented beside the tongue (**Figures 1B, D**, **Supplementary Figure 1**)

### The biodiversity of the pregnant mice microbiome had no difference between RA and control groups

**Figure 2:**
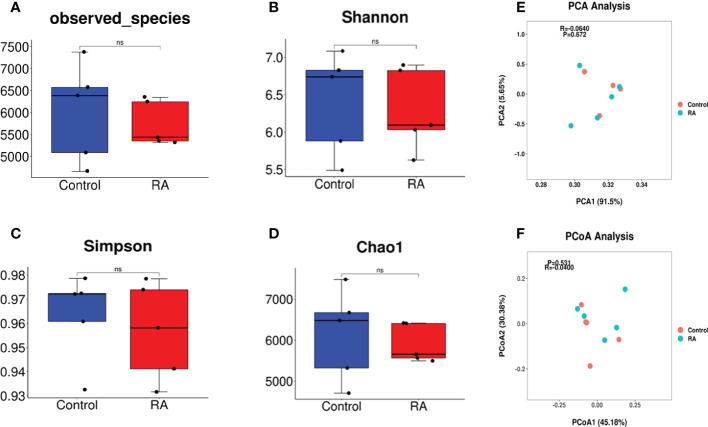
Gut microbiome diversity and structure analysis. **(A-D)** Alpha diversity differences between the RA and control groups were estimated by the observed species**(A)**, Shannon **(B)**, Simpson **(C)**, and Chao1 **(D)** indices. NS, not significant. **(E, F)** Beta diversity differences between the RA and control groups were estimated by the PCA (R=-0.0640, P=0.672) **(E)** and PCoA (R=-0.04, P=0.531) **(F)** of the microbiota (P and R value by ANOSIM analysis, -1≤R ≤ 1, p<0.05 were considered statistically significant).

To detect the composition and structure of the microbial community in RA-treated pregnant mice and controls, we conducted the analyses of alpha and beta diversity of the microbiome in the pregnant mice feces samples. Alpha diversity reflects the diversity of intestinal microflora in individuals and does not involve the comparison between individuals. Results on alpha diversity verified that compared with the control group, the indices of observed species, Shannon, Simpson, and Chao1 were no differences in the RA group (**Figures 2A-D**). Beta diversity is adopted to illustrate phylogenetic differences in microbial communities between the diseased and controls. This method can present the bacterial difference between two groups based on the distance. PCA analysis failed to demonstrate the significant difference in distribution between the two groups, with the principal components of 91.5% and 5.65% (**Figure 2E**). The result of PCoA revealed that there was a similar bacterial environment between controls and RA-treated pregnant mice (R=-0.04, P=0.531, **Figure 2F**).

### The expression of *Lactobacillus* was increased in the RA group

**Figure 3:**
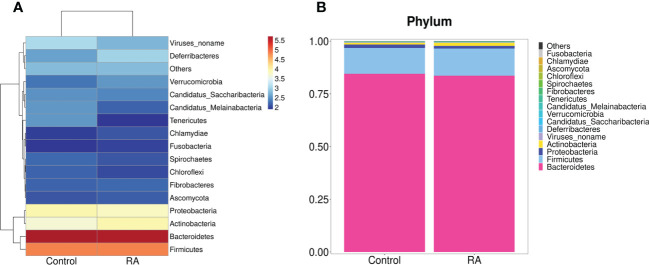
Gut microbiome structure analysis. Component proportions of bacterial phylum in the RA and control groups by heatmap **(A)** and stacked bar **(B)**; n = 5 for the RA group and n = 5 for the control group.

**Figure 4:**
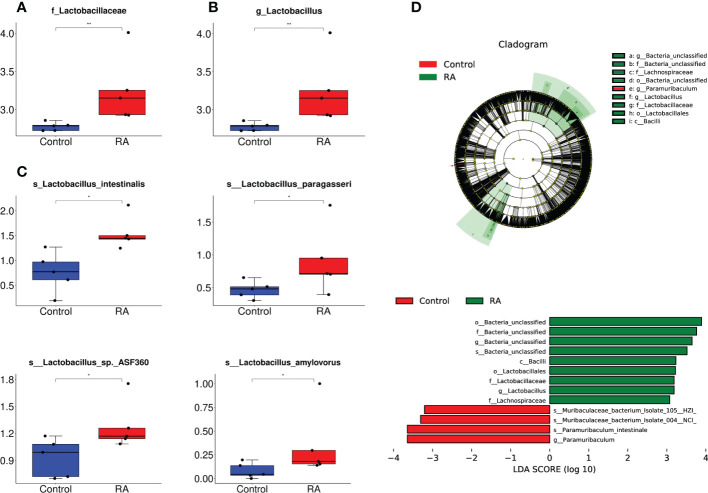
The relative abundance of microbial taxa at the family, genus, and species levels. **(A, B)** The relative abundance of *Lactobacillales* and *Lactobacillaceae* enriched in RA *vs* control at different levels. **(A)**, at family level; **(B)**, at genus level. **(C)** The relative abundance of *Lactobacillus intestinalis*, *Lactobacillus paragasseri*, *Lactobacillus* sp. *ASF360* and *Lactobacillus amylovorus* enriched in RA *vs*. control. The box represents the interquartile ranges, inner line denotes the median. **(D)** The differences in abundance between the RA and control group by LEfSe analysis. o, order; f, family; g, genus; s, specie. *p < 0.05, ** *p* < 0.01 compared with control group.

Subsequently, the relative abundance of microbial taxa at the phylum, class, order, family genus, and species levels were confirmed. Among them, *Bacteroidetes*, *Firmicutes*, *Proteobacteria*, and *Actinobacteria* occupied the main position in both two groups at the phylum level. (**Figures 3A, B**). At the family and genus levels, *Lactobacillales* and *Lactobacillaceae* were the most abundant in the RA group (**Figures 4A, B**), other up-regulation bacteria were listed in **Supplementary Table 3** and **Supplementary Table 4**. At the species level, the expression of *Lactobacillus intestinalis*, *Lactobacillus paragasseri*, *Lactobacillus* sp. *ASF360* and *Lactobacillus amylovorus* were increased in the RA group (**Figure 4C**), other abundant bacteria were listed in **Supplementary Table 5**. To further explore the influence of bacteria on the control and RA group, LEfSe method was used to revealed the influence of significantly different bacteria on the two groups. *Lactobacillales*, *Lactobacillaceae*, and *Lactobacillus* were enriched in the RA-treated group compared with the control (**Figure 4D**).

### Metabolism-related functions played a major role in the RA and control groups

**Figure 5:**
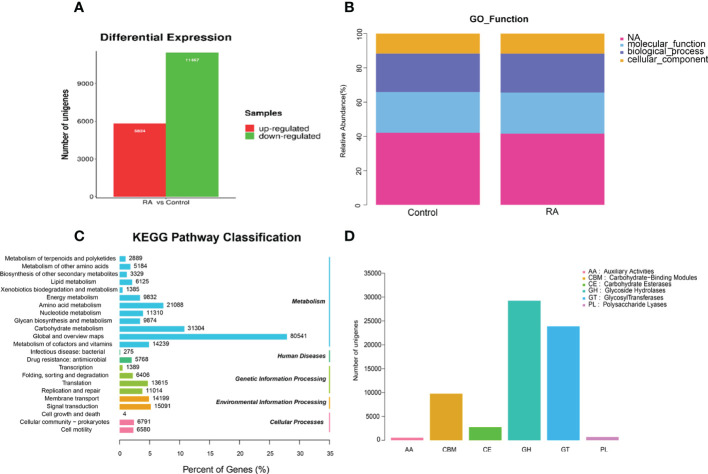
The differential gene expression and gene function in RA and control group. **(A)** Differentially expressed unigenes between RA and control groups. **(B)** Relative abundance of GO function. **(C)** KEGG pathway classification. **(D)** CAZy category.

Next, we investigated the total gene expression of faeces samples in two groups. Compared with the control group, the total gene expression level was changed dramatically under RA treated which affected metabolic pathway *via* gene expression regulation. **Figure 5A** displayed that in RA versus (vs) control group, 5824 unigenes were up-regulated and 11467 were down-regulated in the differentially expressed genes.

The gene ontology (GO) analysis provided controlled vocabularies of defined terms representing gene product properties. Molecular functions and biological processes were clearly influenced by RA compared to the control groups (**Figure 5B**). The results of kyoto encyclopedia of genes and genomes (KEGG) analysis (**Figure 5C**) suggested that metabolism-related pathways took the lead, including global and overview maps, carbohydrate metabolism, and amino acid metabolism. So, we focused on carbohydrate metabolism. Dysregulation of the microbiota caused alterations in carbohydrate-active enzymes (CAZymes), which interfere with carbohydrate metabolism (Onyango et al., 2021). The main role of CAZymes was to generate and break down complex carbohydrates and glycoconjugates, allowing them to exert a huge number of biological effects (Cantarel et al., 2009). CAZymes are primarily studied through the CAZy database. Our experiments illustrated that the order of the proportion of the experimental group and the control group from high to low was glycoside hydrolase (GH) > glycosyltransferase (GT) > carbohydrate-binding module (CBM) > carbohydrate esterifying enzyme (CE) > polysaccharide lyase (PL) > auxiliary redox enzyme (AA) (**Figure 5D**).

### Metabolically related pathways were enriched in the RA and control groups

**Figure 6:**
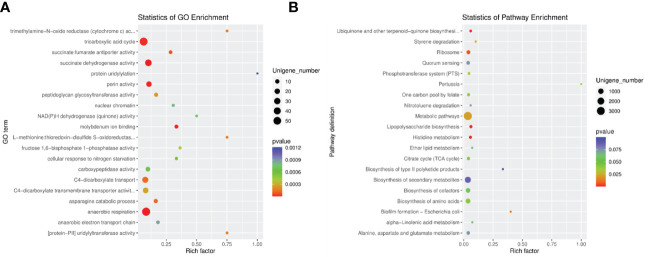
The enrichment analysis in RA and control group. **(A)** GO enrichment analysis of differentially expressed unigenes between RA and control groups. **(B)** Pathway classification based on KEGG enrichment analysis of differentially expressed unigenes between RA and control groups. Rich factor, is the ratio of the number of differentially expressed genes (DEGs) to the number of total genes in this pathway.

Since there was a study found that *Lactobacillus* could cause metabolic disorders (Tokarek et al., 2021), our results detected that *Lactobacillus* enriched in the RA-treated group compared with the control, and metabolism-related pathways were the most differentially expressed pathways between two groups by KEGG, we then analyzed the relevant pathways which manifested the changes in the digestive tracts in order to further investigate the relationship between microbiota and metabolism in both RA-induced and control pregnant mice. We integrated the function information related to each gene in GO and KEGG databases. In GO enrichment analysis, tricarboxylic acid (TCA) cycle, succinate dehydrogenase activity, porin activity, and anaerobic respiration enriched in both RA and control groups (**Figure 6A**); In KEGG enrichment analysis, ribosome, metabolic pathways, lipopolysaccharide biosynthesis, and histidine metabolism enriched in both RA and control groups (**Figure 6B**). Analysis of differential gene function (GO, CAZy, and KEGG pathway) and gene set enrichment analysis (GO and KEGG pathway) manifested significant changes in carbohydrate metabolism and energy metabolism in both the RA and control groups. The reason might be due to the number of *Lactobacillus* changes that could cause metabolic disorders, which might play a key role in the potential interaction effects between CP in fetal mice and gut microbiome in pregnant mice.

### The amount of lactate, product of *Lactobacillus*, was up-regulated in pregnant mice plasma, amniotic fluid and fetal palatal tissue

**Figure 7:**
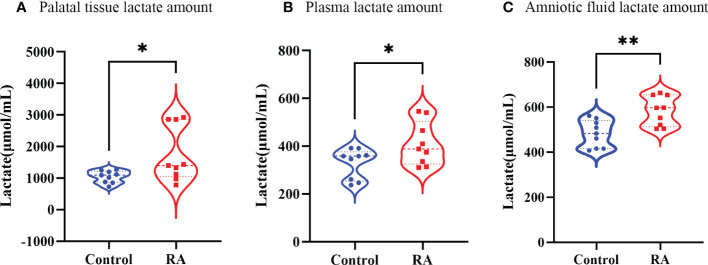
The lactate amount in RA and control group. **(A)** The lactate amount in palate tissue between RA and control groups (n=3). **(B)** The lactate amount in plasma between RA and control groups (n=3). **(C)** The lactate content in amniotic fluid between RA and control groups (n=3). *p < 0.05, ** *p* < 0.01 compared with control group.

As a product of *Lactobacillus*, lactate amount might reflect metabolic function of *Lactobacillus* (Mikelsaar et al., 2016). Thus, we tested lactate amount in plasma, amniotic fluid, and palatal tissues. Plasma and amniotic fluid were extracted from E16.5 pregnant mice. Palatal tissues were extracted from E16.5 fetal mice. It was investigated lactate amount was up-regulated in palatal tissue, plasma, and amniotic fluid in the RA group (**Figures 7A-C**), which were consist with our metagenomic sequencing analysis.

## Discussion

The gut microbiota was one of the causative factors affecting metabolic syndrome. It acted as a critical part in regulating dietary fat absorption and lipid metabolism by influencing bile acid metabolism, producing short-chain fatty acids, and regulating the intestinal endocrine system (Yu et al., 2019). Current research indicated that the administration of RA to pregnant sows ameliorated developmental defects in Hoxa1^-/-^ fetal pigs, and maternal RA administration restored bacterial ecological dysbiosis in Hoxa1^-/-^ neonates and altered the bacterial composition of the small intestine in non-Hoxa1^-/-^ neonates (Zhou et al., 2021). However, it remains unclear whether fetal CP induced by RA is related to the maternal gut microbiome without genetic disorders.As one of the main drugs causing CP, RA was reported to increase the relative abundance of *Lactobacillus* spp. in the gut (Abdelhamid and Luo, 2018). Consistently, in our research, we firstly set up the RA-induced CP model in fetal mice whose palate shelves failed to elevate into a horizontal position completely, which was accordant with previous reports (Campbell et al., 2004; Dong et al., 2017; Gao et al., 2017; Wang et al., 2017; Peng et al., 2020), then we found that the expression of *Lactobacillus* was significantly increased in the RA group, including *Lactobacillus intestinalis*, and *Lactobacillus* sp. *ASF360*, *Lactobacillus paragasseri*, and *Lactobacillus amylovorus*. These results indicated that the formation of fetal cleft palate might associate with the excess increase of *Lactobacillus* in the gut microbiota of RA-treated pregnant mice.

It is well-known that a major characteristic of *Lactobacilli* is their ability to metabolize glycogen-derived products under anaerobic conditions to produce lactate (Witkin and Linhares, 2017). That might the path that affected the whole body. A retrospective observational study showed that various peripartal risk factors (e.g., uterine rupture, placental abruption, chorioamnionitis, and pre-eclampsia) might have contributed to higher lactate values and that lactate levels in maternal cord blood were associated with a mixed metabolic acidosis in the fetus after birth (Gaertner et al., 2021). Also, maternal lactate and umbilical arterial and venous lactate concentrations were significantly higher in intrauterine growth-retarded infants compared with normal infants (Marconi et al., 1990). A further novel finding was that, as an end product of glycolysis, lactate had a controlling function in the fate of mouse embryonic stem cells (Tian and Zhou, 2022). In our results, it was notable that the production of lactate was up-regulated in both pregnant mice and fetal mice treated with RA.

Studies have found that measuring molecular types in maternal and fetal blood, as well as in the fetal brain, revealed that when gut flora was deficient during pregnancy, specific metabolites were often also reduced or absent, which subsequently affected fetal brain development (Vuong et al., 2020). In addition, maternal gut microbiota was associated with offspring metabolic phenotype. During pregnancy, the SCFAGPR41 and SCFA-GPR43 axes could pass on the mother’s gut microbiota to offspring to make them resistant to obesity. GPR41 and GPR43 in the sympathetic nerve, intestinal tract, and pancreas of the embryo could sense SCFAs in the maternal gut microbiota, thereby affecting the prenatal development of the metabolic and neural system (Kimura et al., 2020). Our research also detected that the increase of *Lactobacillus* in the gut of pregnant mice in the RA group promoted lactate amount in the palatal tissue of fetal mice *via* the “gut-plasma-amniotic fluid” pathway, which then caused cleft palate. However, the specific mechanism still needs to be further investigated.

Metagenomics is an effective way to clarify the relationship between gut microbiome and pathogenesis. In our work, to further explore the mechanisms involved in CP formation between pregnant mice and fetuses, the GO and KEGG enrichment analysis implied that metabolic-related pathways were significantly enriched in the gut microbiome of pregnant mice, including metabolic pathways, TCA cycle, anaerobic, and so on. Different sequencing results revealed an association between *Lactobacillus* and metabolism. Functional proteomics and metaproteomics showed that *Lactobacillus* altered metabolic pathways (e.g., carbohydrate transport and metabolism, pyruvate metabolism, proteolytic system, amino acid metabolism, and protein synthesis) to a large extent (De Angelis et al., 2016). 16SRNA results also showed a correlation between *Lactobacillus* and glycolysis enzyme (Brandt and Barrangou, 2018). In addition, an earlier study on chicken and mouse embryos confirmed that energy metabolism was tightly regulated during development (Oginuma et al., 2020). Mouse early preimplantation embryos did not rely on glucose as their primary energy source, but participated in the TCA cycle and produce ATP using pyruvate and lactate (Bénazéraf et al., 2010). An important metabolic shift occurs during embryo implantation, resulting in increased glucose uptake and enhanced glycolysis activity. At this point, most of the glycolysis activity co-exists with an active TCA cycle and oxidative phosphorylation, causing the production of lactic acid. However, with the formation of organs, the intense glycolysis activity of embryos declined, and respiration became the main way of energy generation (Henrique et al., 2015; Oginuma et al., 2017). CAZy analysis demonstrated that the percentage of GH and GT family enzymes in the control group and experimental group were higher than other enzymes. GT and GH play important roles in the formation of glycosylation (Cantarel et al., 2009). In some genetic disorders, individuals had abnormalities in glycosylation caused by genetic mutations that could lead to a variety of symptoms, including epilepsy, cleft palate, and heart defects (Lukacs et al., 2019). A report showed that in the process of mammalian organ formation, golgin subfamily B member 1 (Golgb1) mutant embryos caused cleft palate in mice, which was due to reduce hyaluronan accumulation and impair protein glycosylation in the palatal mesenchyme (Lan et al., 2016). All of these results indicated that the formation of CP by RA was related to metabolism, and the maternal environment also affected the development of the fetal palate.

To sum up, our results suggest that RA-induced maternal gut microbiome alterations in Lactobacillus cause lactate variation in embryo palate shelves which may affect fetal palate development through metabolic change. This study has some limitations. Firstly, the number of mice samples of each group was five. If more samples were included the results might be more in-depth. Secondly, we didn’t validate the sequencing results directly as we can’t find the commercial *Lactobacillus* which was increased in the RA group. That was why we measured the amount of lactate, the product of *Lactobacillus*, in pregnant mice plasma, amniotic fluid, and fetal palatal tissue to confirm the results indirectly. Further studies about microbiome and metabolism on CP are needed to fully clarify which we focus on now. And it will be better that the relationship between human maternal gut microbiome and CP can be explored if available.

## Data availability statement

The datasets presented in this study can be found in online repositories. The names of the repository/repositories and accession number(s) can be found in the article/**Supplementary Material**.

## Ethics statement

The animal study was reviewed and approved by Animal Care and Use Committee of the School of Stomatology, Capital Medical University (Beijing, China, permit number: KQYY-202109-006).

## Author contributions

YW: Conceptualization, Methodology, Investigation, Software, Data analysis, Writing - review & editing. JC: Investigation. XW: Investigation. CG and XP: Formal analysis; Writing - review & editing. YL: Supervision; Writing - review & editing. TL: Formal analysis; Resources; Supervision. JD: Conceptualization; Funding acquisition; Project administration; Supervision; Roles/Writing - original draft. All authors contributed to the article and approved the submitted version.

## Supplementary material

Supplementary Figure 1 The morphology of palatal shelves and tongue at E16.5 embryos. **(A)** The morphology of palatal shelves and tongues in control group. **(B)** The morphology of palatal shelves and tongues in RA group. PS, palatal shelf; T, tongue; NS, nasal septum; magnification ×40, scale bar 200 μm.
