# Supplementary figures and images for: Novel investigations in retinoic-acid-induced cleft palate about the gut microbiome of pregnant mice
Source: Front Cell Infect Microbiol. 2022 Dec 15;12:1042779. doi: 10.3389/fcimb.2022.1042779 (PMC9798234; doi:10.3389/fcimb.2022.1042779)

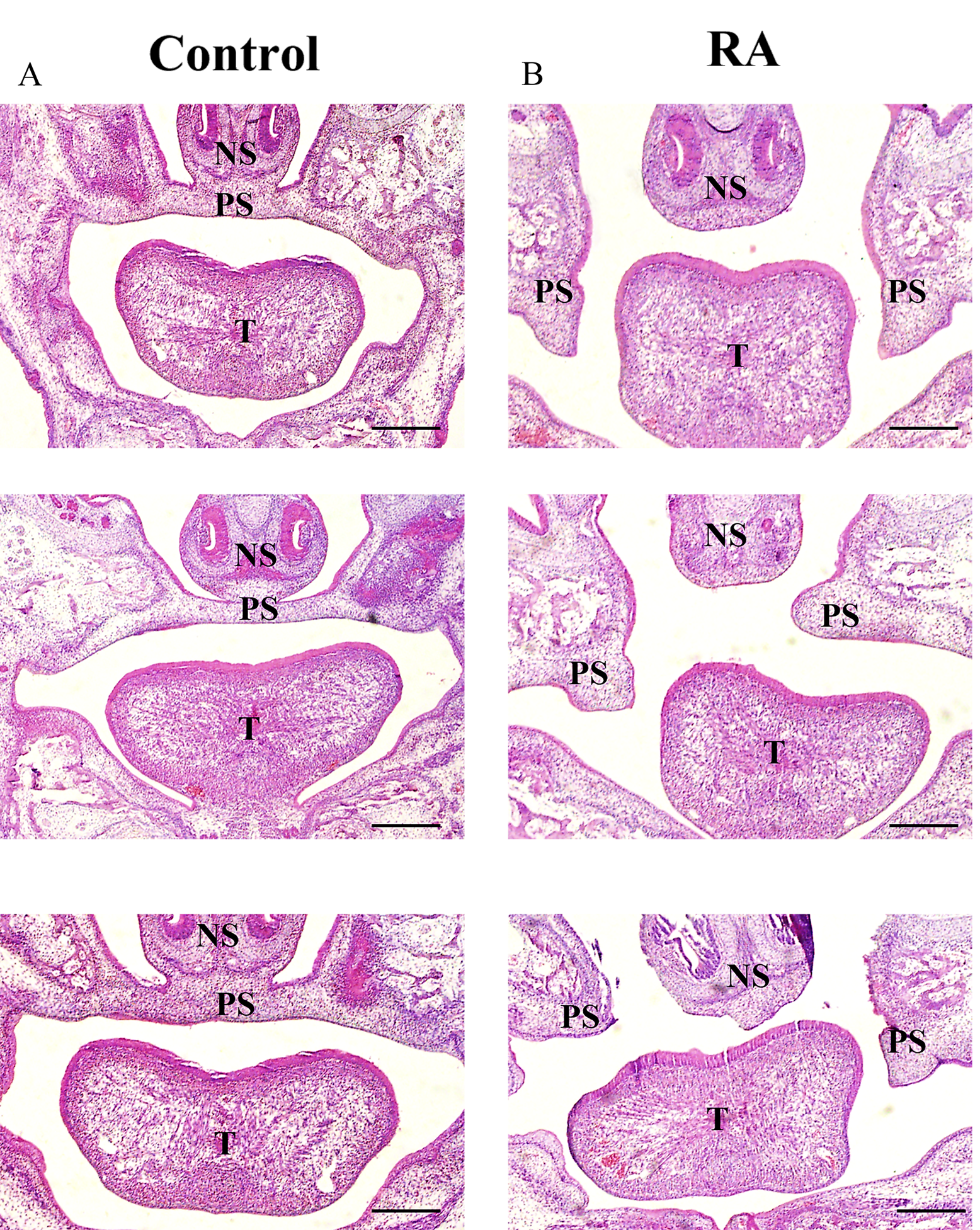

Supplement: Supplementary Figure 1 — The morphology of palatal shelves and tongue at E16.5 embryos. (A) The morphology of palatal shelves and tongues in control group. (B) The morphology of palatal shelves and tongues in RA group. PS, palatal shelf; T, tongue; NS, nasal septum; magnification ×40, scale bar 200 μm. [file Image_1.tif]
