# Supplementary material for: Novel investigations in retinoic-acid-induced cleft palate about the gut microbiome of pregnant mice
Source: Front Cell Infect Microbiol. 2022 Dec 15;12:1042779. doi: 10.3389/fcimb.2022.1042779 (PMC9798234; doi:10.3389/fcimb.2022.1042779)
Supplement: Supplementary file 2 [file Table_1.docx]

Supplementary Table 1 The procedures and software of data analysis

| Analysis item | Software | Versions |
| --- | --- | --- |
| Removal of sequencing adapters | cutadapt | 1.9 |
| Trim low-quality reads | fqtrim | 0.94 |
| Data quality control statistics | FastQC | 0.10.1 |
| Removal of host contamination | Bowtie2 | 2.2.0 |
| construct the metagenome | IDBA-UD | 1.1.1 |
| Statistical evaluation of assembly results | QUAST | 3.2 |
| Comparison of database | DIAMOND | 0.9.14 |
| Species annotation | ACGT101_metagenome | 2.0 |
| Function annotation | ACGT101_metagenome | 2.0 |
