# Supplementary material for: Novel investigations in retinoic-acid-induced cleft palate about the gut microbiome of pregnant mice
Source: Front Cell Infect Microbiol. 2022 Dec 15;12:1042779. doi: 10.3389/fcimb.2022.1042779 (PMC9798234; doi:10.3389/fcimb.2022.1042779)
Supplement: Supplementary file 3 [file Table_2.docx]

Supplementary Table 2 The database information

| Database name | Version/Date | Link |
| --- | --- | --- |
| NR | nr_meta_20191121 | <ftp://ftp.ncbi.nlm.nih.gov/blast/db/FASTA/nr.gz> |
| GO Database | go_2018.12.21 | http://geneontology.org/ |
| KEGG Database | KEGG-release_87.1 | http://www.genome.jp/kegg/ |
| CAZy Database | CAZy-2019.07.3 | http://www.cazy.org/ |
