# Supplementary material for: Novel investigations in retinoic-acid-induced cleft palate about the gut microbiome of pregnant mice
Source: Front Cell Infect Microbiol. 2022 Dec 15;12:1042779. doi: 10.3389/fcimb.2022.1042779 (PMC9798234; doi:10.3389/fcimb.2022.1042779)
Supplement: Supplementary file 4 [file Table_3.docx]

Supplementary Table 3 Different abundance at family level between two groups

| Family | mean RA | mean Control | regulation | p value | significance |
| --- | --- | --- | --- | --- | --- |
| f__LactobaAcillaceae | 3034.83 | 602.18 | up | 0.01 | yes |
| f__Leptospiraceae | 3.20 | 1.21 | up | 0.03 | yes |
| f__Desulfomicrobiaceae | 52.58 | 20.17 | up | 0.05 | yes |
