# Supplementary material for: Novel investigations in retinoic-acid-induced cleft palate about the gut microbiome of pregnant mice
Source: Front Cell Infect Microbiol. 2022 Dec 15;12:1042779. doi: 10.3389/fcimb.2022.1042779 (PMC9798234; doi:10.3389/fcimb.2022.1042779)
Supplement: Supplementary file 5 [file Table_4.docx]

Supplementary Table 4 Different abundance at genus level between two groups

| Genus | mean RA | mean Control | regulation | p value | significance |
| --- | --- | --- | --- | --- | --- |
| g__Lactobacillus | 3035.94 | 600.65 | up | 0.01 | yes |
| g__Thermodesulfitimonas | 3.00 | 0.46 | up | 0.01 | yes |
| g__Leminorella | 0.43 | 0.06 | up | 0.03 | yes |
| g__Caenibacillus | 24.81 | 1.75 | up | 0.03 | yes |
| g__Leptospira | 3.20 | 1.21 | up | 0.03 | yes |
| g__Siphonobacter | 3.24 | 0.91 | up | 0.03 | yes |
| g__Desulfohalotomaculum | 1.54 | 0.48 | up | 0.03 | yes |
| g__Anaeroglobus | 0.62 | 0.12 | up | 0.05 | yes |
| g__Desulfitibacter | 7.82 | 3.20 | up | 0.05 | yes |
| g__Sediminispirochaeta | 2.26 | 0.50 | up | 0.05 | yes |
| g__Arsenicibacter | 1.34 | 0.49 | up | 0.05 | yes |
