# Supplementary material for: Novel investigations in retinoic-acid-induced cleft palate about the gut microbiome of pregnant mice
Source: Front Cell Infect Microbiol. 2022 Dec 15;12:1042779. doi: 10.3389/fcimb.2022.1042779 (PMC9798234; doi:10.3389/fcimb.2022.1042779)
Supplement: Supplementary file 6 [file Table_5.docx]

Supplementary Table 5 Different abundance at genus level between two groups

|  | |  |  |  |  |
| --- | --- | --- | --- | --- | --- |
| Species | meanRA | mean  Control | regula-tion | p  value | signific-ance |
| s__Dehalobacter_sp._MCB1 | 0.64 | 0.04 | up | 0.01 | yes |
| s__Clostridium_sp._AM25-23AC | 19.58 | 9.63 | up | 0.01 | yes |
| s__Megasphaera_elsdenii | 10.24 | 3.49 | up | 0.01 | yes |
| s__Erysipelotrichaceae_bacterium_3_1_53 | 8.01 | 3.01 | up | 0.01 | yes |
| s__Sphaerochaeta_sp. | 5.89 | 1.83 | up | 0.01 | yes |
| s__Lysinibacillus_varians | 5.11 | 0.86 | up | 0.01 | yes |
| s__Desulfitibacter_sp._BRH_c19 | 3.16 | 0.72 | up | 0.01 | yes |
| s__Tepidanaerobacter_acetatoxydans | 2.95 | 0.83 | up | 0.01 | yes |
| s__Thermodesulfitimonas_autotrophica | 3 | 0.46 | up | 0.01 | yes |
| s__Bacillus_oryziterrae | 1.35 | 0.47 | up | 0.01 | yes |
| s__Methanobacterium_sp._MB1 | 0.91 | 0.28 | up | 0.01 | yes |
| s__Veillonellaceae_bacterium_DNF00626 | 0.62 | 0.14 | up | 0.01 | yes |
| s__Oceanobacillus_limi | 0.54 | 0.21 | up | 0.01 | yes |
| s__Chryseobacterium_carnipullorum | 0.22 | 0.07 | up | 0.01 | yes |
| s__Lactobacillus_intestinalis | 45.54 | 6.89 | up | 0.02 | yes |
| s__Blautia_sp._AF13-16 | 6.3 | 1.39 | up | 0.02 | yes |
| s__Clostridium_sp._AF37-5AT | 4.23 | 1.78 | up | 0.02 | yes |
| s__Leminorella_grimontii | 0.43 | 0.06 | up | 0.03 | yes |
| s__Aneurinibacillus_sp._XH2 | 0.28 | 0.05 | up | 0.03 | yes |
| s__Campylobacter_sp._RM6137 | 0.29 | 0.04 | up | 0.03 | yes |
| s__Lactobacillus_unclassified | 286.82 | 35.16 | up | 0.03 | yes |
| s__Caenibacillus_caldisaponilyticus | 24.81 | 1.75 | up | 0.03 | yes |
| s__Roseburia_inulinivorans_CAG:15 | 7.11 | 3.32 | up | 0.03 | yes |
| s__Ruminococcus_sp._AM58-7XD | 6.51 | 3.17 | up | 0.03 | yes |
| s__Coprococcus_sp._AF16-22 | 5.44 | 2.43 | up | 0.03 | yes |
| s__Bacteroidetes_bacterium_GWE2_29_8 | 4.89 | 1.99 | up | 0.03 | yes |
| s__Ruminococcus_sp._AF14-5 | 4.54 | 1.35 | up | 0.03 | yes |
| s__Bifidobacterium_thermophilum | 4.23 | 1.26 | up | 0.03 | yes |
| s__Siphonobacter_sp._BAB-5385 | 3.24 | 0.91 | up | 0.03 | yes |
| s__Desulfohalotomaculum_alkaliphilum | 1.54 | 0.48 | up | 0.03 | yes |
| s__Bifidobacterium_scardovii | 1.29 | 0.25 | up | 0.03 | yes |
| s__Sedimentibacter_sp._SX930 | 0.98 | 0.31 | up | 0.03 | yes |
| s__Candidatus_Atribacteria_bacterium_1244-E10-H5-B2 | 0.75 | 0.1 | up | 0.03 | yes |
| s__Xanthomonas_campestris | 0.41 | 0.11 | up | 0.04 | yes |
| s__Geobacter_uraniireducens | 0.35 | 0.06 | up | 0.04 | yes |
| s__Streptococcus_downei | 0.3 | 0.04 | up | 0.04 | yes |
| s__Planctomycetes_bacterium_Pla123a | 1.08 | 0.06 | up | 0.05 | yes |
| s__Anaeroglobus_geminatus | 0.62 | 0.12 | up | 0.05 | yes |
| s__Chlamydia_abortus | 76.88 | 33.69 | up | 0.05 | yes |
| s__Ruminococcaceae_bacterium_P7 | 40.67 | 12.02 | up | 0.05 | yes |
| s__Lactobacillus_sp._ASF360 | 22.17 | 8.4 | up | 0.05 | yes |
| s__Lactobacillus_paragasseri | 14.73 | 2.05 | up | 0.05 | yes |
| s__Ruminococcus_sp._OM07-7 | 7.43 | 2.55 | up | 0.05 | yes |
| s__Sporanaerobacter_sp._PP17-6a | 7.55 | 1.71 | up | 0.05 | yes |
| s__Peptostreptococcaceae_bacterium_VA2 | 3.62 | 1.56 | up | 0.05 | yes |
| s__Faecalibacterium_sp._AF10-46 | 2.52 | 0.54 | up | 0.05 | yes |
| s__Caloramator_sp._ALD01 | 2.16 | 0.61 | up | 0.05 | yes |
| s__Lactobacillus_amylovorus | 2.26 | 0.23 | up | 0.05 | yes |
| s__Paenibacillus_terrae | 1.63 | 0.76 | up | 0.05 | yes |
| s__Candidatus_Cryosericum_hinesii | 1.67 | 0.2 | up | 0.05 | yes |
| s__Arsenicibacter_rosenii | 1.34 | 0.49 | up | 0.05 | yes |
| s__Sediminispirochaeta_smaragdinae | 1.37 | 0.33 | up | 0.05 | yes |
| s__Bacillus_sp._FJAT-42376 | 1.1 | 0.53 | up | 0.05 | yes |
| s__Gilliamella_apicola | 0.88 | 0.32 | up | 0.05 | yes |
| s__Ruminococcus_sp._AF19-29 | 0.66 | 0.1 | up | 0.05 | yes |
